# The role of elastosonography, gray-scale and colour flow Doppler sonography in prediction of malignancy in thyroid nodules

**DOI:** 10.2478/raon-2014-0007

**Published:** 2014-11-05

**Authors:** Idil Gunes Tatar, Aydin Kurt, Kerim Bora Yilmaz, Mehmet Doğan, Baki Hekimoglu, Sema Hucumenoglu

**Affiliations:** 1 Department of Radiology, Ankara Diskapı Training and Research Hospital, Ankara, Turkey; 2 Department of General Surgery, Ankara Diskapi Training and Research Hospital, Ankara, Turkey; 3 Department of Pathology, Dr. Abdurrahman Yurtaslan Ankara Oncology Training and Research Hospital, Ankara, Turkey; 4 Ankara Research and Training Hospital, Department of Pathology, Ankara, Turkey

**Keywords:** ultrasound, Doppler, elastosonography, thyroid, malignancy

## Abstract

**Background:**

Ultrasound is as a noninvasive method commonly used in the work-up of thyroid nodules. This study aimed to evaluate the usefulness of sonographic and elastosonographic parameters in the discrimination of malignancy.

**Patients and methods.:**

150 thyroid nodules were evaluated by gray-scale, Doppler and elastosonography. The cytological analysis revealed that 141 nodules were benign and 9 were malignant.

**Results:**

Orientation of the nodule was the only sonographic parameter associated with malignancy (p = 0.003). In the strain ratio analysis the best cut-off point was 1.935 to discriminate malignancy (p = 0.000), with 100% sensitivity, 76% specificity, 100% negative predictive value, 78.5% positive predictive value and 78% accuracy rate. There was a statistically significant correlation between the elasticity score and malignancy (p = 0.001). Most of the benign nodules had score 2 and 3, none of them displayed score 5. On the other hand, none of the malignant nodules had score 1 and 2, most of them displaying score 5.

**Conclusions:**

A change in the diagnostic algorithm of the thyroid nodules should be considered integrating the elastosonographic analysis.

## Introduction

Thyroid nodules are recognised if palpated by the patient, during physical examination or by a radiological assessment. Studies reveal that 2.7–17% of the thyroid nodules are malignant whereas the majority is benign.^1^ The best way to differentiate between malignant and benign thyroid nodules is the cytological evaluation of the material sampled by fine-needle aspiration (FNA).^2^ Considering the high prevalence of thyroid nodules, it is not feasible to evaluate all thyroid nodules by FNA. In contemporary guidelines, ultrasound (US) evaluation is recommended as a noninvasive method for the management of thyroid nodules.^3^–^5^ US is the main tool in the risk analysis of both palpable and non-palpable thyroid nodules and their selection for FNA cytology (FNAC).^6^ Nevertheless, it has some limitations in differentiating between benign and malignant thyroid nodules resulting from the inconsistency of sensitivity, specificity, positive and negative predictive values of sonographic features in the published studies.^7^

Malignant thyroid nodules are usually stiff on palpation.^8^ Stiffness of the nodule is determined by its cellularity and can be detected by ultrasound elastography (USE). USE, which was first suggested by Ophir *et al*., analyses the elasticity of a nodule by measuring the amount of distortion which takes place when the nodule is compressed.^9^–^12^ When compression is applied to the thyroid tissue, it produces the strain which is defined as the displacement of tissue in vertical direction, and the amount of strain is bigger in softer tissue compared to harder tissue. There are two kinds of evaluations performed by USE. One is based on the elasticity scores (ES) and the other is based on the strain ratio (SR) measurements.

The aim of this study is to evaluate the usefulness of gray-scale and colour flow Doppler US parameters, ES and SR in the differentiation of benign versus malignant thyroid nodules.

## Patients and methods

**FIGURE 1.**
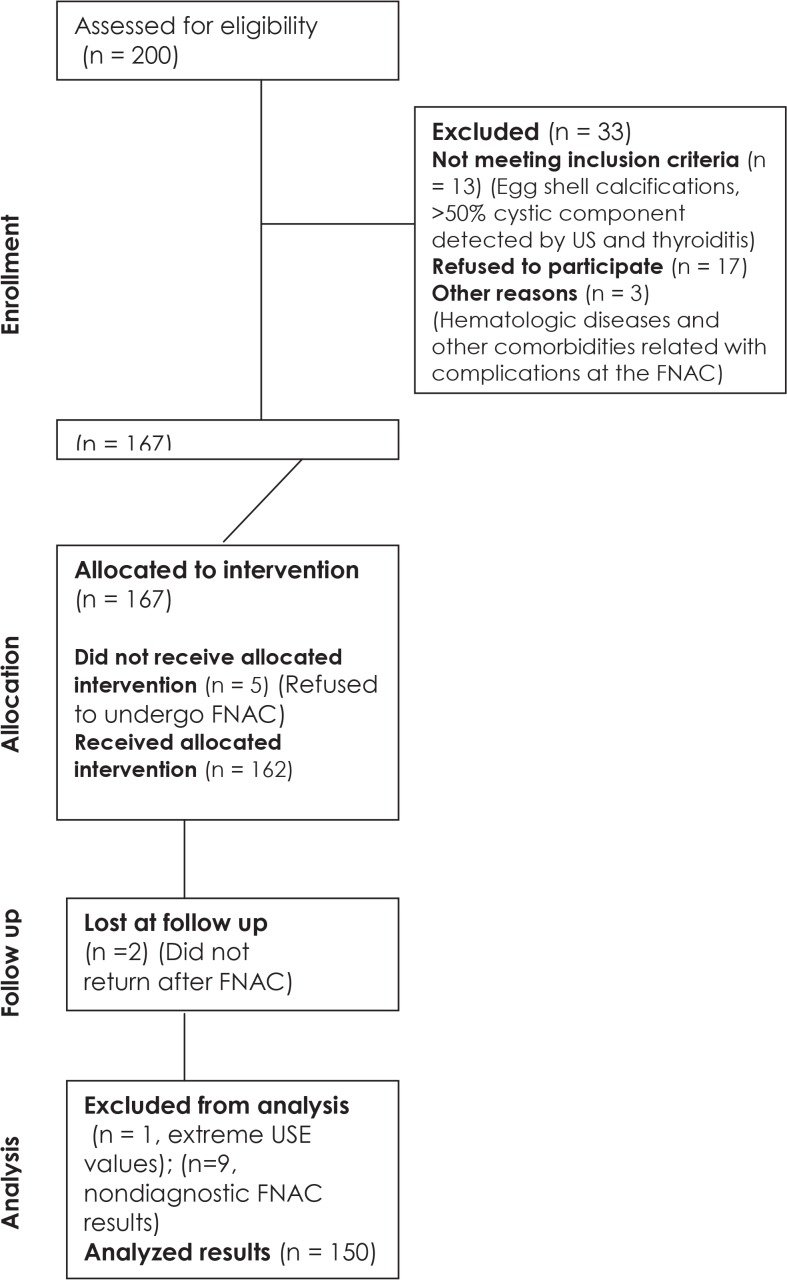
Patients included in the study.

In total 200 patients with thyroid nodules who were referred to the radiology department for FNAC by the general surgery department were assessed for eligibility. After obtaining the approval of the institutional ethical board committee, informed consent was taken from the participants. Seventeen patients refused to participate; furthermore, 13 patients with nodules having egg shell calcifications, >50% cystic component detected by US and thyroiditis, three patients with hematologic diseases and other comorbidities related with complications at the FNA were excluded from the study. From the remaining 167 patients 5 patients refused to undergo FNAC. The study sample included 162 patients (mean age 49 years; range, 20–83 years; 132 females and 30 males). Patients were examined by US and USE prior to FNAC. The patient flow of the study is summarized in Figure 1.

All patients were examined by conventional US and USE using a linear transducer 8–13 MHz (Logos EUB 8500; Hitachi, Tokyo, Japan). All sonographic examinations were conducted by a sonographer with 15 years of sonography experience. The patients were requested to lie down in the supine position with the neck slightly extended. Carotid arteries were avoided if it was possible.

Gray-scale images were first obtained for each nodule. The following US parameters were evaluated: location (right lobe, left lobe, isthmus), internal structure (solid, cystic, mixed), echo structure (hyperechoic, isoechoic, or hypoechoic compared to normal thyroid parenchyme), margin (smooth, microlobulated, irregular), presence or absence of calcifications (none, microcalcifications which are defined as hyperechoic spots small than 2 mm, macrocalcifications, mixed), orientation (parallel, nonparallel), and halo sign (presence or absence).

Colour flow Doppler patterns were defined as type 1: no internal blood flow, type 2: perinodular blood flow and absent or little internal blood flow and type 3: significant internal and absent or little perinodular blood flow.

An intermittent light pressure was applied until the pressure was standardized until a sinusoid was formed between two predetermined lines to maintain the pressure at the optimal level. Sonograms and elastograms were displayed next to each other for the identification of the nodule. The nodule and surrounding thyroid parenchymal tissue was evaluated.

A region-of-interest (ROI) box with an adjustable size covering the majority of the nodule was placed (average strain represented as A) taking the adjacent normal thyroid parenchymal tissue preferably which has the same depth and the same size as the reference (average strain represented as B). Strain ratio (B/A) which reflects the stiffness of the lesion was calculated for each nodule.

An elastogram based on a colour scale was displayed on the B-mode image which ranges from red to blue. Red colour represents tissues with greatest elasticity meaning softest components, whereas blue colour represents tissue with no strain meaning hardest components. Each nodule was given an ES based on a five-point scale developed by Rago *et al*.^13^ ES 1 indicated homogenously elastic nodule. ES 2 indicated predominantly elastic nodule. ES 3 indicated elasticity only at the periphery of the nodule. ES 4 indicated absence of intranodular elasticity. ES 5 indicated absence of elasticity in the nodule or in the posterior shadowing. In the literature nodules having scores 4 or 5 were classified as suspicious for malignancy.

FNA was done under US guidance by the same radiologist at the end of the sonographic examination. Half of the aspirate was dried in the air, and the rest was fixed with alcohol and stained with Papanicolaou and Giemsa. The materials were interpreted by an experienced cytologist. The sufficiency of material was determined according to the guidelines of the Papanicolaou Society.^14^ The pathologist was blind to the sonographic findings.

To eliminate false-positive results, 9 patients with cytologic malignancies underwent surgery. The surgical material was fixed with formalin, embedded in paraffin and was stained by hematoxylin and eosin.

Fisher Exact Chi-Square test was used for categorical data analysis and Mann Whitney-U test for numerical data analysis. Receiver operating characteristic curve analysis was used to calculate the best cut-off value of the SR. Sensitivity, specificity, and accuracy rate, positive predictive value and negative predictive value were calculated for SR calculations. P values less than 0.05 were accepted as significant.

## Results

Among 162 patients who underwent FNAC, two patients were lost during the follow-up. Nine patients with nondiagnostic FNAC results and one patient with extreme USE results were excluded from the study. Finally the statistical analysis was done on 150 patients. According to FNAC results 141 of the nodules were benign. Six nodules had malignant cytology results and pathology proved to be papillary carcinoma in five of them and follicular carcinoma in the remaining one. Three nodules had undetermined cytology findings and pathology proved to be papillary carcinoma in one of them and follicular carcinoma in the other two.

Sex and age of the patients were not associated with malignancy with p values being equal to 0.562 and 0.571, respectively.

### Gray-scale US features associated with malignancy

Location, structure, echogenicity, margin, presence or absence of calcifications and halo sign, vascularity of the nodule were not related to malignancy. Orientation of the nodule was the only sonographic parameter associated with malignancy (p = 0.003). In addition, 93.6% of the benign nodules had parallel orientation whereas 55.6% of the malignant nodules displayed parallel orientation (i.e. transverse diameter greater than vertical diameter).

**FIGURE 2.**
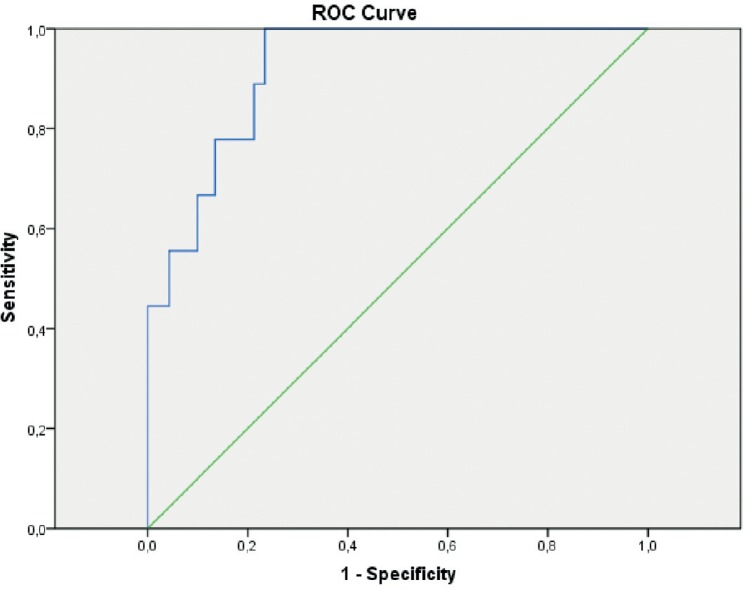
ROC curve analysis of the strain ratio measurements of thyroid nodules by elastosonography.

SR is calculated by dividing the mean strain of the adjacent normal thyroid parenchymal tissue by the mean intranodular strain. By using the receiver operating characteristic analysis, the best cut-off point was found to be 1.935 to differentiate benign from malignant nodules with 95% confidence interval (p = 0.000, AUC = 0.920) (Figure 2). The sensitivity, specificity, negative predictive value, positive predictive value and accuracy rate of the SR analysis were 100%, 76%, 100%, 78.5% and 78% respectively.

**FIGURE 3.**
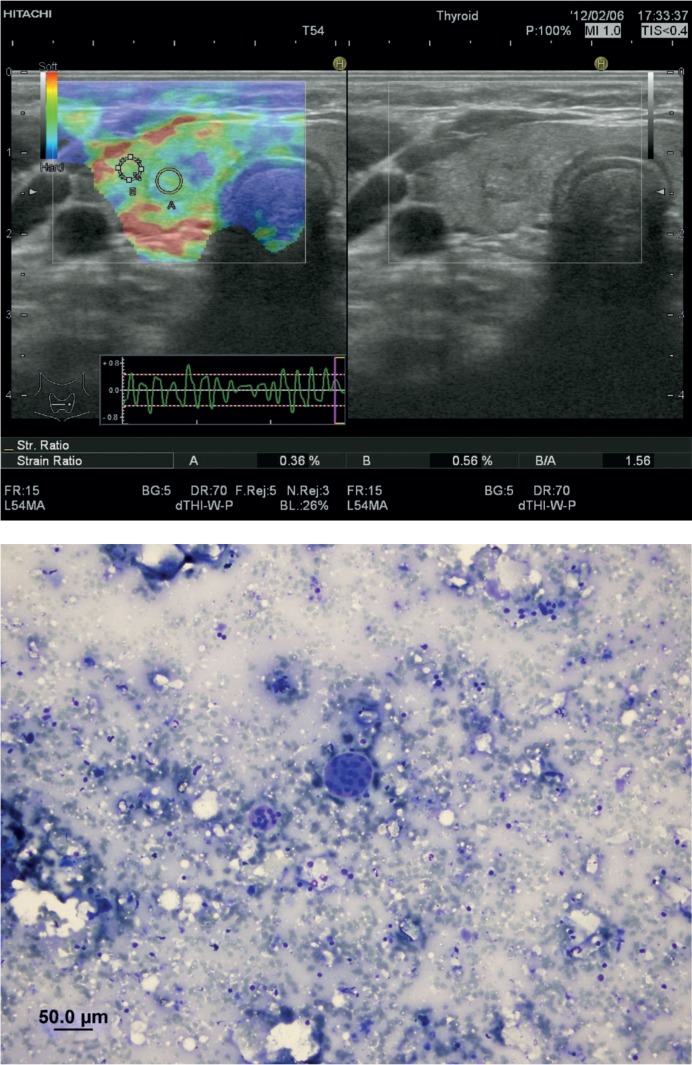
**A** Elastosonographic evaluation of an isoechoic solid nodule measuring 6.7 × 5.4 mm with well-defined margins, parallel orientation and peripheral halo in the right lobe of the thyroid gland of a 44 year-old female patient. Region of interest of the nodule is represented by A, region of interest of the adjacent thyroid parenchyme is represented by B. Elasticity score interpretation was 2, strain ratio measurement was 1.56. **B** Fine needle aspiration cytology revealed benign thyrocyte group forming low cellularity macrofollicule formation (May-Grunwald giemsa stain, X200).

**FIGURE 4.**
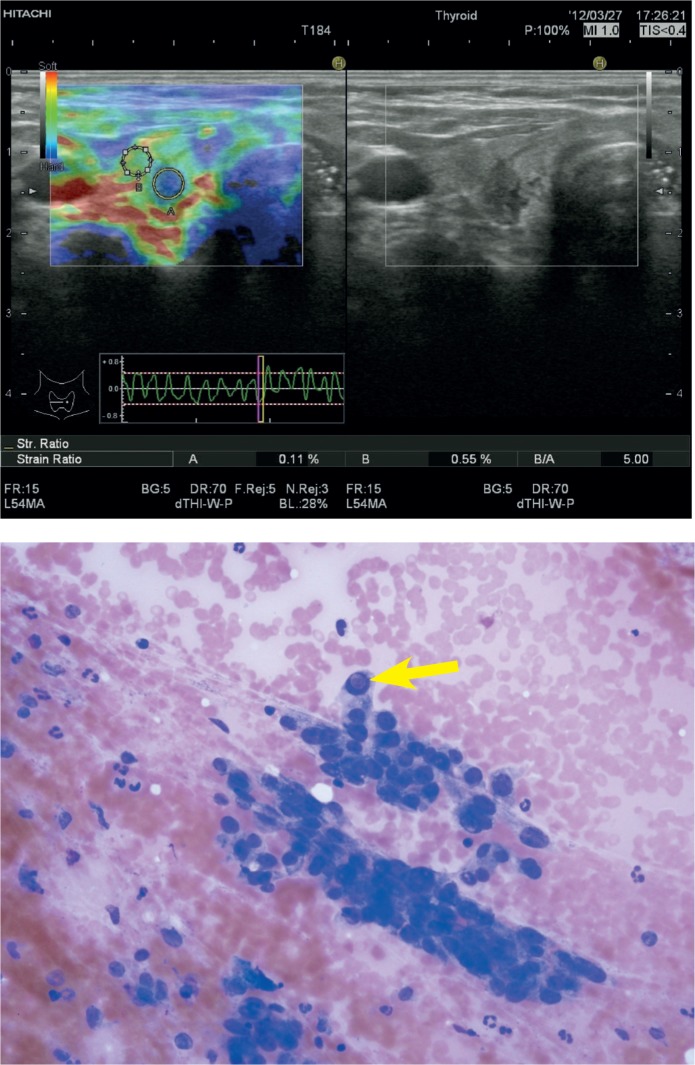
**A** Elastosonographic evaluation of a hypoechoic, solid nodule measuring 6 × 8 mm with irregular margins, antiparallel orientation in the right lobe of the thyroid gland of a 53 year-old female patient. Region of interest of the nodule is represented by A, region of interest of the adjacent thyroid parenchyme is represented by B. Elasticity score interpretation was 4, strain ratio measurement was 5. **B** Fine needle aspiration cytology revealed papillary carcinoma cells with atypical nuclei containing intranuclear inclusion bodies (arrow) (May-Grunwald giemsa stain, ×400). Histopathology proved to be papillary carcinoma.

There was a statistically significant correlation between the ES and cytology of the nodules (p = 0.001). When the elasticity scores of the benign nodules were analysed most of the benign nodules had ES 2 and 3. Among the benign nodules 2 nodules displayed ES 1 (1.4%); 69 nodules showed ES 2 (48.9%) (Figure 3); 56 nodules had ES 3 (39.7%) and 14 nodules revealed ES 4 (9.9%). None of the benign nodules displayed ES 5. On the other hand, none of the malignant nodules had ES 1 and 2, most of them displaying an ES 5. Among the malignant nodules 2 nodules displayed ES 3 (22.2%); 3 nodules had ES 4 (33.3%) (Figure 4); 4 nodules showed ES 5 (44.4%).

## Discussion

The literature asserts that sonographic features and elastosonographic evaluation have variable diagnostic performances in the discrimination of benign and malignant nodules. In a retrospective multicenter study nonparallel orientation, spiculated margin, marked hypoechogenicity, microcalcification and macrocalcification were found to be associated with malignancy.^15^ Yet, Iannuccilli *et al*., declared that the presence of intrinsic microcalcification, which is defined as a “snowstorm” pattern of calcification which has 100% specifity for malignancy, was the only statistically significant feature associated with malignancy.^7^

The use of USE for improving the diagnostic accuracy of sonographic examination of the thyroid nodules was first described by Lyshchik *et al.*^16^ Authors prospectively evaluated the role of USE for discriminating malignancy and found that SR value greater than 4 had the highest association with malignancy (p <0.001); with 96% specificity and 82% sensitivity.^16^

In a recent meta-analysis published by Razavi *et al*., which evaluated twenty four studies USE was concluded to be more sensitive and specific compared to each of the ultrasound features.^17^ In the retrospective research conducted by Moon *et al*., USE alone and the combination of USE and gray-scale US, demonstrated inferior performance in the differentiation of malignant versus benign thyroid nodules compared to gray-scale US features.^18^ Other studies have also demonstrated that USE is superior to US or the addition of USE to US, increased the diagnostic performance of US findings was higher.^19^,^20^ Bojunga *et al*., carried out a meta-analysis of USE studies for the discrimination of malignant thyroid nodules. USE exhibited a mean sensitivity of 92% and a specificity of 90% for the diagnosis of malignant thyroid nodules. A wide range of specificity was noted in different studies. Authors explained that USE could be used with high sensitivity in the management of thyroid nodules and might be a useful tool in conjunction with or even instead of FNAC for the selection of patients for surgery.^21^

In the literature various cut-off values have been suggested for the discrimination of malignancy in SR analysis. Ning *et al*., concluded that the best cut-off point of SR was 4.2 for the discrimination of malignancy.^22^ In the study of Xing *et al*., the best cut-off values for large and small nodules were different, i.e. for nodules that are larger than 1 cm, the cut-off value was 3.98 whereas for the ones equal or smaller than 1 cm the cut-off value was 4.21. Authors also concluded that SR analysis demonstrated better diagnostic performance compared to the 4 scale ES method.^23^ Kagoya *et al*. indicated that SR value greater than 1.5 was found as the predictor of malignancy, with 90% sensitivity and 50% specificity.^24^

In our study, the distribution of the strain ratio value confirmed that benign nodules are much softer than the malignant ones and the best cut-off value was found to be 1.925. We have also established a statistically significant correlation between the ES and pathology of the nodules. None of the benign nodules demonstrated ES 5 and none of the malignant nodules showed ES 1 and 2. Antiparallel orientation was the only gray-scale US feature associated with malignancy. In the light of the results; taller than wide shape, ES 3, 4 and especially 5, SR greater than 1.925 were associated with malignancy. Presence of at least one of these findings should prompt the FNAC analysis in the primary evaluation of the thyroid nodules.

USE can also be beneficial in the management of patients with FNAC with nondiagnostic or indeterminate results, which constitute the major limitation of FNAC of thyroid nodules. A nondiagnostic cytology can be obtained in cystic or hemorrhagic lesions due to lack of adequate number of cells for diagnosis. In 10 to 20% of all FNA materials, although the collected material is insufficient, cytology is classified as indeterminate, meaning that differentiation between follicular adenoma and follicular carcinoma or the follicular variant of a papillary thyroid carcinoma is not made.^25^ Among patients with FNA resulting in indeterminate lesion, 25% end up with a final diagnosis of malignancy on histology.^26^

Rago *et al*., carried out a study to investigate the role of USE in the presurgical diagnosis of nodules with indeterminate or nondiagnostic cytology.^27^ For indeterminate lesions the sensitivity and specificity of USE were 96.8% and 91.8%, respectively. For nodules with nondiagnostic cytology USE had a sensitivity of 87.5% and a specificity of 86.7%. Authors showed that USE could be a valuable method for the discrimination of malignancy in nodules with indeterminate or nondiagnostic cytology and it eventually contributes to the selection of patients for surgery. USE evaluation of such lesions can not only help decrease the unnecessarily use of FNAC but also increases the diagnostic ability. In diagnostic algorithm of the clinicians crucial problems such as repeating biopsies and delays in diagnosis can be prevented with the help of USE.

A limitation of our study is the low prevalence of malignant nodules. Larger prospective studies are needed to confirm our results. For the nodules with indeterminate or nondiagnostic cytology a study with a larger cohort is necessary to evaluate the role of USE in the selection of patients for surgery.

## Conclusions

Nonparallel orientation of the nodule can be used as a gray-scale US criterion indicative of malignancy. In the USE evaluation elasticity scores 3, 4 and especially 5; strain ratio measurements greater than 1.925 are suggestive of malignancy.

A change in the diagnostic algorithm of the thyroid nodules should be considered integrating the USE analysis. Accordingly, in the diagnostic work-up of the thyroid nodules, USE evaluation should take part in the standard protocol in clinical practice for the decision and evaluation period of the treatment.
